# Comparative Transcriptome Analysis Reveals a Preformed Defense System in Apple Root of a Resistant Genotype of G.935 in the Absence of Pathogen

**DOI:** 10.1155/2017/8950746

**Published:** 2017-03-30

**Authors:** Yanmin Zhu, Jonathan Shao, Zhe Zhou, Robert E. Davis

**Affiliations:** ^1^USDA-ARS, Tree Fruit Research Laboratory, Wenatchee, WA 98801, USA; ^2^USDA-ARS, Molecular Plant Pathology Laboratory, Beltsville, MD 20705, USA; ^3^Tree Fruit Research Institute, Chinese Academy of Agricultural Sciences, Xingcheng, Liaoning 10081, China

## Abstract

Two apple rootstock genotypes G.935 and B.9 were recently demonstrated to exhibit distinct resistance responses following infection by* Pythium ultimum.* As part of an effort to elucidate the genetic regulation of apple root resistance to soilborne pathogens, preinoculation transcriptome variations in roots of these two apple rootstock genotypes are hypothesized to contribute to the observed disease resistance phenotypes. Results from current comparative transcriptome analysis demonstrated elevated transcript abundance for many genes which function in a system-wide defense response in the root tissue of the resistant genotype of G.935 in comparison with susceptible B.9. Based on the functional annotation, these differentially expressed genes encode proteins that function in several tiers of defense responses, such as pattern recognition receptors for pathogen detection and subsequent signal transduction, defense hormone biosynthesis and signaling, transcription factors with known roles in defense activation, enzymes of secondary metabolism, and various classes of resistance proteins. The data set suggested a more poised status, which is ready to defend pathogen infection, in the root tissues of resistant genotype of G.935, compared to the susceptible B.9. The significance of preformed defense in the absence of a pathogen toward overall resistance phenotypes in apple root and the potential fitness cost due to the overactivated defense system were discussed.

## 1. Introduction

During their coevolution with pathogens, plants developed a sophisticated, two-layered innate immune system that confers resistance against most microbial pathogens [1–3]. The first line of defense is initiated upon perception of a conserved pathogen-associated molecular pattern (PAMP) by pattern recognition receptor (PRR) embedded in the plant's plasma membrane, activating so-called PAMP-triggered immunity (PTI). PTI is generally considered as a basal, nonspecific response [1]. However, adapted pathogens are able to counteract the basal resistance of a host through the secretion of evolved molecules called “effector” proteins, which suppress or bypass PTI [4–6]. On the plant side, coevolved resistance (R) genes directly or indirectly detect pathogen virulence effectors and initiate the second layer of defense called effector-triggered immunity (ETI) [1, 7]. The second layer of plant immunity relies on the specific recognition of pathogen effectors by disease resistance proteins (R), leading to a strong and specific immune response toward those isolates of a pathogen that produce the recognized effector. These common responses of plant defense systems occur after the plant encounters a pathogen, but is it possible that in some cases plant defenses are already “primed” before inoculation?

The genus* Pythium*, which consists of over 100 species, is ubiquitously distributed and capable of long-term survival in soil by producing thick-walled oospores. Germination of phytopathogenic* Pythium* spp. oospores initiates infections of seeds or roots [8].* P. ultimum* is one of its most significant plant pathogens from this genus and infection by this pathogen results in damping off or root rot, which leads to plant wilting, reduced yield, and mortality on many economically important crops [9].* P. ultimum* is also identified as a major component in the pathogen complex which incited apple replant disease [10]. A recent study using two apple rootstock genotypes demonstrated that G.935 is more resistant, and B.9 is more susceptible, to infection by* P. ultimum* [11]. In the present study, as part of an effort to elucidate the genetic regulation of apple root resistance to soilborne pathogens, it was hypothesized that preinoculation transcriptome variation(s) in roots contributes to genotype-specific disease resistance.

To test this hypothesis, a comparison of global transcriptional landscape in the root tissues of two apple rootstock cultivars (B.9 and G.935) before inoculation was carried out using RNA-seq based transcriptome sequencing; the two cultivars were recently demonstrated to exhibit distinct resistance responses following infection by* P. ultimum* [11]. The results of our study revealed that the defense system of the more highly disease resistant apple rootstock genotype G.935 was poised at an elevated level prior to intentional inoculation with a pathogen. The finding is reminiscent of the recent report of so-called preformed defense or the elevated and constitutive expression of plant defense genes contributing to genotype-specific disease resistance among rice genotypes [12]. The present observations and those of others [12, 13] raise questions concerning the mechanisms that are responsible for elevation of defense systems before inoculation.

## 2. Materials and Methods

### 2.1. Preparation by Tissue Culture Procedure and Maintenance of Plant Materials

Tissue culture based micropropagation procedures were used to obtain cloned plants for both apple rootstock genotypes as described previously [11]. To minimize the influence of nongenetic factors, a synchronized micropropagation procedure was employed to generate plants of equivalent developmental stage for both B.9 and G.935. Specifically, the root tissues of 4-week-old plants after root induction in tissue culture medium were then transferred to pasteurized (in an oven at 85°C for two consecutive overnights) Sunshine™ potting mix soil (SUN GRO Horticulture Ltd, Bellevue, WA) for a period of one week with “in-soil” acclimation in a growth chamber to further the differentiation of root tissues. To minimize transplanting effects from tissue culture medium to soil condition, a transparent 7′′ Vented Humidity Dome (Greenhouse Megastore, Danville, IL) was placed on top of a 10 × 20-inch flat tray holding the pots for retention of humidity. Root tissues were collected by flash freezing in liquid nitrogen and stored at −80°C before RNA isolation.

### 2.2. Total RNA Isolation and High-Throughput mRNA Sequencing

Total RNA isolation followed the method previously described in Zhu et al., 2016 [11]. Root tissues of both resistant G.935 and susceptible B.9 were represented by three biological replicates, and each replicate included the pooled root tissues from three plants. The frozen root tissue samples were ground to a fine powder in liquid nitrogen, and RNA quantity was determined using a Nanodrop spectrophotometer (ND-1000; Thermo Fisher Scientific). The RNA integrity number (RIN) was evaluated using an Agilent 2100 Bioanalyzer. Only RNA with an RIN value of *x* ≥ 8 was used for RNA-seq. Oligo (dT) magnetic beads were utilized to isolate poly-(A) tails containing mRNAs from total RNA and then fragmentation buffer was added to interrupt mRNA to short fragments. Using these short fragments as templates, first-strand cDNA was synthesized using reverse transcriptase and a random hexamer primer. Second-strand cDNA fragments were then synthesized using a buffer, DNA polymerase I, dNTPs, and RNase H. After purification and paired-end (PE) repair protocols were performed, the cDNA fragments were ligated to sequencing adapters and amplified using PCR to obtain the final PE cDNA library. The library preparation and RNA-sequencing with 150 bp paired-end (PE) were completed at the Center for Genome Research and Biocomputing in Oregon State University using an Illumina HiSeqTM 3000 (Illumina Inc., San Diego, CA, USA).

### 2.3. Mapping of Sequence Reads and Differentially Expressed Gene Analysis

Reads from B.9 (susceptible) and G.935 (resistant) libraries were mapped to the nucleotide sequences of predicted coding genes of the* Malus* x* domestica* Whole Genome v3.0.a1 (https://www.rosaceae.org/analysis/162) using the ultrafast, memory-efficient short read aligner Bowtie 2-2.2.5 which utilizes a Burrows-Wheeler index [14]. Count data were obtained for each coding sequence. Estimation and statistical analysis of expression levels using count data of each gene with 3 replicates for each library was performed using the DEseq2 package [15] and R x64 3.3.1 program (https://www.r-project.org/). Differentially expressed genes (DEGs) were identified by comparing transcript abundance between B.9 and G.935 root transcriptome data set with the cutoff values of Log_2_ fold change ≥ 2; and + indicated higher transcript level in the root of G.935 over that in B.9; − indicated the opposite. The annotation of these genes was carried out by BLASTP [13] against NR (nonredundant protein sequences) database and a BLAST database containing genomic sequences for* Arabidopsis (Arabidopsis thaliana)*, corn* (Zea mays)*,* Medicago truncatula*, rice* (Oryza sativa)*, and tomato* (Solanum lycopersicum)*.

### 2.4. Validation of the Expression Pattern of Identified DEGs by qRT-PCR

The same total RNAs that were used for RNA-seq experiments were also used for RNA-seq data validation by qRT-PCR. The total RNA was treated with DNase I (Qiagen, Valencia, CA) and then purified with RNeasy cleanup columns (Qiagen, Valencia, CA). Two *μ*g of DNase-treated RNA was used to synthesize first-strand cDNA using SuperScript™ II reverse transcriptase (Invitrogen, Grand Island, NY) and poly dT (Operon, Huntsville, AL) as the primer. The cDNA was diluted 20 times and 0.6 *μ*L aliquot was used in a 15 *μ*L quantitative PCR (qPCR) reaction mix: 0.45 *μ*L SYBR Green I dye (Invitrogen, Grand Island, NY), 1x iTaq buffer (Biorad, Hercules, CA), 0.2 mM dNTP (Applied Biosystems, Waltham, MA), 2.5 mM MgCl_2_, 0.3 units of iTaq DNA polymerase (Biorad, Hercules, CA), and 0.2 *μ*M forward/reverse primer (IDT, Coralville, IA). Real-time qPCR amplification and detection was conducted using an iQ5 real-time qPCR detection system (Biorad Lab, Hercules, CA) and the following protocol: cycle conditions of 3 min at 95°C and 40 cycles of 10 s at 95°C and 30 s at 59°C. Dissociation curves were run for all of the primers used in this study to determine the presence of any nonspecific amplification. The relative gene expression was measured using the lowest Ct value as the calibrator. “No reverse transcriptase” and “no template” negative controls were included in PCR amplification. Each sample was represented by two independent total RNA isolations converted into two separate cDNAs. Each cDNA sample included two replicates for PCR reactions. Therefore, four separate PCR amplifications (four replicates) were performed on each sample. PCR amplification was carried out in triplicate in a 96-well plate. The target gene expression was normalized to that of the internal reference gene* (MdActin)* using the 2^−ΔΔCT^ method (the comparative Ct method) [16].

## 3. Results

### 3.1. Statistic of Read Mapping and DEG Identification

The numbers of raw reads by RNA-seq and alignment rate for three biological replicates from both B.9 and G.935 genotypes were shown in Table 1. Approximately two-thirds of the predicted CDs (Malus_x_domestica.v3.0.a1_gene_set_cds.fasta.gz) were shown to have reads that mapped. Log_2_FC values of 2 and greater were used as the criteria for differentially expressed gene (DEG) identification, and about 3.01% of all predicted genes in apple genome were identified as DEGs between these two genotypes. Upregulation denotes the higher transcript level in the roots of G.935; conversely, downregulation indicates the lower transcript level in G.935 compared that in B.9. The functional annotation for these DEGs were assigned based on the searching the NR database and the* Arabidopsis*, tomato, corn, rice, poplar, and* Medicago* protein sequences in GenBank.

### 3.2. DEGs with Annotated Function of Defense Hormone Biosynthesis and Signaling

Previously, two apple rootstock cultivars, B.9 and G.935, have demonstrated distinct resistance response to* Pythium ultimum* [11] and* Rhizoctonia solani* (Zhu, unpublished data). Ethylene and jasmonic acid (JA) are well known to be the defense hormones in combating necrotrophic pathogens [17, 18]. As shown in Table 2, in the root tissues without exposure to the pathogen, several DEGs with annotated functions of ethylene biosynthesis and signaling were identified. Two “1-aminocyclopropane-1-carboxylate synthase” (ACS) encoding genes and three “1-aminocyclopropane-1-carboxylate oxidase” (ACO) encoding genes showed considerable higher level of detected transcripts in G.935 than in B.9. Two of three “lipoxygenase” encoding genes, which function in JA biosynthesis pathway, also showed more abundant transcripts in G.935 than in B.9 root tissue. Three out of five genes encoding ERFs (ethylene response factors), which play roles in integrating ET/JA signals, were shown to be with higher expression level in the roots of G.935 as compared with those of B.9. DEGs for gibberellin (GA) signaling and biosynthesis appear to be differentially expressed in the root tissues of two cultivars. There were three receptor GID1 encoding genes showing higher transcript level in G.935 roots compared to that in B.9. On the other hand, two DEGs encoding “gibberellin 2-beta-dioxygenase,” which is involved in GA biosynthesis, were either up- or downregulated. The higher transcript levels for both “abscisic acid 8′-hydroxylase” encoding genes, which involve abscisic acid (ABA) metabolism, were detected in the roots of G.935. Overall, the elevated transcriptional activities related to defense hormone metabolism and signaling were shown to exist in the root tissue of the more resistant genotype of G.935 than in the roots of the susceptible apple rootstock genotype of B.9.

### 3.3. DEGs with Annotated Function of Pattern Recognition Receptors (PRRs) or Receptor with Defense Implication

DEGs encoding several categories of plant PRRs were identified based on root transcriptome comparison between two apple rootstock genotypes (Table 3; Supplementary File number 1 in Supplementary Material available online at https://doi.org/10.1155/2017/8950746). LysM domain receptors are known to be able to bind PAMPs such as chitin from fungal pathogens [19, 20] and detect the presence of pathogens. The higher transcript levels of four LysM domain receptors encoding genes were detected in the roots of G.935 as compared to those from B.9. Two DEGs encoding “BRASSINOSTEROID INSENSITIVE1-associated receptor kinase 1-like receptors” showed significantly higher level of detected transcripts in G.935 than in B.9. A total of 15 WAK (wall associated kinase) or WAK-like encoding genes were shown to be differentially expressed between the root tissues from two cultivars, although 9 out of 15 of them exhibited lower transcript levels in G.935. Notably, more than half of these DEGs encode WAK-like 16 homologs.

### 3.4. Lectin Receptor Kinase, Glutamate Receptor Encoding Genes, and Other Receptor Kinases

Lectin-domain containing receptor kinases are known to participate in immune response and resistance signal transduction in other pathosystems [21, 22]. Between the root tissues for two apple rootstock cultivars, ten “L-type lectin-domain containing receptor kinase” and 25 “G-type lectin S-receptor-like serine/threonine-protein kinase” encoding genes showed differential expression patterns (Table 4). Most of these receptor kinase-encoding genes exhibited higher transcript abundance in the resistant cultivars G.935. Fourteen “glutamate receptor” encoding genes were identified as DEGs between two cultivars; and most (9 out of 14) of them showed the elevated expression levels in the roots of G.935 as compared to B.9. The biological function of glutamate receptor is not well understood, but preliminary data point to its roles in defense response [23, 24]. Additionally, there were many DEGs which were annotated as LRR receptor-like serine/threonine-protein kinase, receptor-like protein kinase, receptor-like protein, and cysteine-rich receptor-like protein kinase. Double or triple the numbers of DEGs showed higher transcript levels in the root tissues of G.935, in contrast to relatively small number of genes with the lower transcript levels (Table 5; Supplementary File number 1).

### 3.5. DEGs with the Annotated Function of Oxidation and Reduction Processes

The balance of ROS production for the purpose of defense signaling and the scavenging capacity to avoid cellular damage could be critical in plant immunity [25]. A total of 27 DEGs encoding proteins involved with the oxidation and reduction process were identified from transcriptome comparison between these two cultivars, including “glutathione S-transferase,” “peroxidase,” and “superoxide dismutase” (Table 6). Except for the group of identified DEGs encoding superoxide dismutase, the vast majority of DEGs from the other two groups showed lower expression levels in root tissue of G.935 than in that of B.9.

### 3.6. DEGs with the Annotated Function as Transcription Factors (TF)

**Figure 1:**
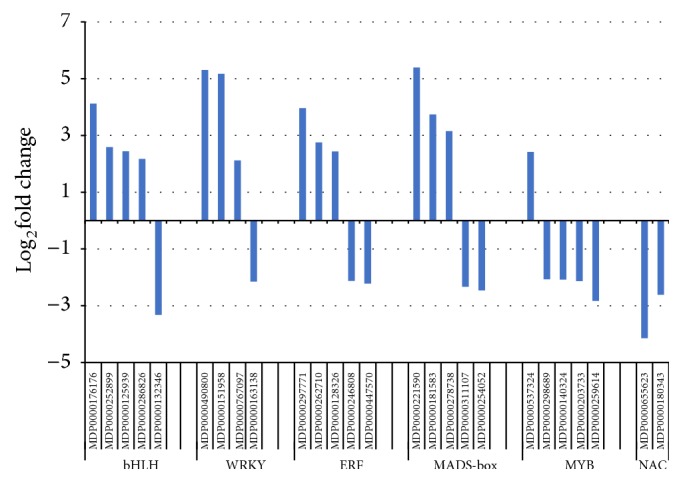
Identified DEGs encoding TF families implicated in defense responses. Gene reference numbers and the TF family names were shown on the *x*-axis; Log_2_FC values were indicated on the *y* axis, where positive values mean the higher transcript level in the roots of G.935 over that of B.9; the opposite is for the negative Log_2_FC values.

Transcription factors (TFs) are the key regulator of cellular transcriptional activities, and several TF families are well known for their roles in defense response such as MYB (myeloblastosis oncogene), ERF (ethylene response factor), and WRKY (containing signature WRKY amino acid residues) families [26, 27]. From this data set DEGs encoding proteins belonging to several families of TFs showed considerable variation at the expression levels between two cultivars (Figure 1). The comparative transcript abundance demonstrated specific patterns for each TF family. For example, most bHLH, WRKY, and ERF-encoding genes showed higher transcript level in the roots of G.935, but a majority or all of identified MYB and NAC encoding DEGs showed opposite patterns with higher transcript level in the roots of B.9.

### 3.7. DEGs Annotated as Resistance Genes

**Figure 2:**
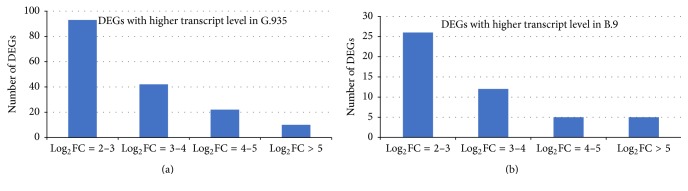
The number and the range of Log_2_FC values for identified R-genes with higher expression levels in G.935 (a) and B.9 (b). For both cultivars, more genes exhibited moderate transcript level variation with Log_2_FC at 2-3. Relative small number showed significant variations of transcript abundance as compared with other cultivar. Detailed information was shown in Supplementary File.

**Figure 3:**
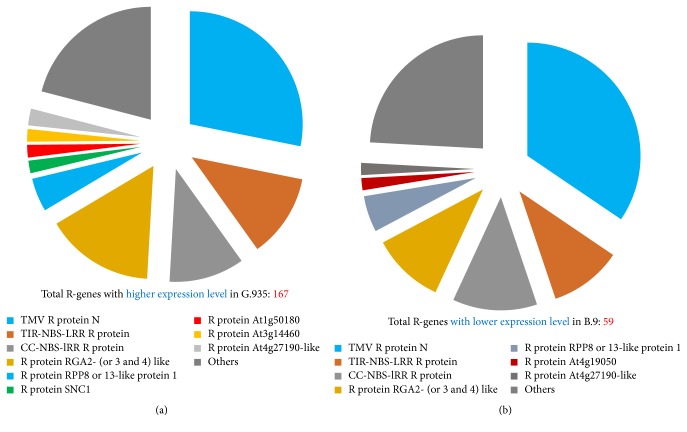
Classification for identified DEGs encoding resistance proteins. An overall similarity for the subcategories of R protein encoding genes which are with higher expression level in either B.9 or G.935 was observed. However, three times more DEGs were identified from G.935 as compared to that from B.9. A few subcategories R protein encoding genes were unique in each cultivar such as R protein SNC1 from G.935 and R protein At4g19050 from B.9.

**Figure 4:**
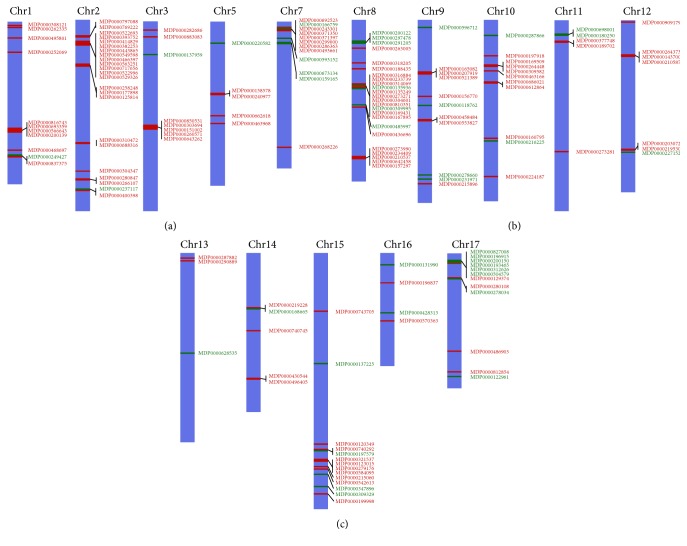
Chromosome locations of identified R-genes. The numbers on top of purple bars denote various apple chromosomes. MDP numbers highlighted in red indicate those with higher transcript level in the resistant cultivar G.935; MDP numbers highlighted in green indicate lower transcript level in G.935. Some of the identified R-genes have not localized to a specific chromosome.

A large number of R-genes were identified as DEGs by comparing root transcriptome between the resistant cultivar G.935 and the susceptible cultivar B.9. As shown in Figures 2(a) and 2(b) (and see details in Supplementary File number 1), fifty-nine identified R-genes showed higher expression level in susceptible B.9 (than in G.935); in contrast, 167 DEGs showed elevated transcript levels in the roots of the resistant cultivar G.935; the number of identified DEGs is almost 3 times larger than those identified from B.9. However, the distribution pattern among subcategories of annotated R-gene based on Log_2_FC values is comparable. According to their functional annotation, it appears that similar subcategories of R protein encoding genes were identified, with higher transcript level in either cultivar (Figures 3(a) and 3(b)). “TMV resistance protein N-like” R-genes are the most prevalent group, with 28% and 34% of all identified R-genes showing higher expression level in G.935 and B.9, respectively. CC-NBS-LRR resistance protein encoding genes, TIR-NBS-LRR resistance protein encoding genes, and disease resistance protein RGA2 (or 3, 4, and 8) are the other major subcategories which contain the double-digit percentage to total identified R-genes. Three small groups of DEGs, those encoded proteins similar to “SUPPRESSOR of npr1-1, CONSTITUTIVE 1-like,” “R protein At1g50180,” and “R protein At3g14460,” were identified only from G.935 as DEGs with higher transcript levels. In contrast, two small groups, “At4g19050 R protein” and “At4g27190-like R protein” encoding genes were identified only from B.9 roots with higher transcript levels. The chromosomal locations of these R-genes showed the typical clustered pattern of R-gene distribution along most of the 17 apple chromosomes (Figure 4).

### 3.8. DEGs Encoding PR Proteins, Cell Wall Metabolism, and Secondary Metabolism

As shown in Table 7, genes encoding four classes of pathogenesis-related (PR) proteins showed differential expression patterns between two cultivars, but the patterns varied for each class. Two genes encoding “pathogenesis-related protein 1-like” and four out of five identified “thaumatin-like protein” encoding genes showed higher expression level in G.935; conversely, four “chitinase” genes and three “glucan endo-1,3-beta-glucosidase” genes all exhibited lower expression level in G.935. All 11 cell wall metabolisms related genes except one in three groups, that is, “pectin esterase inhibitor,” “polygalacturonase,” and “cellulose synthase-like protein” encoding genes, demonstrated the higher expression level in G.935, as compared with that in B.9. Plant secondary metabolisms were well-demonstrated to be a part of induced defense response in many pathosystems [28–31]. Although DEGs encoding proteins belonging to at least thirteen gene families of enzymes with the implication of functioning in plant secondary metabolisms were identified, nevertheless, between two cultivars a relative comparable status was observed in terms of the number of DEGs showing either higher or lower transcript levels (Table 8; Supplementary File number 1).

### 3.9. Validation of the Expression Patterns of Selected DEGs by qRT-PCR

**Figure 5:**
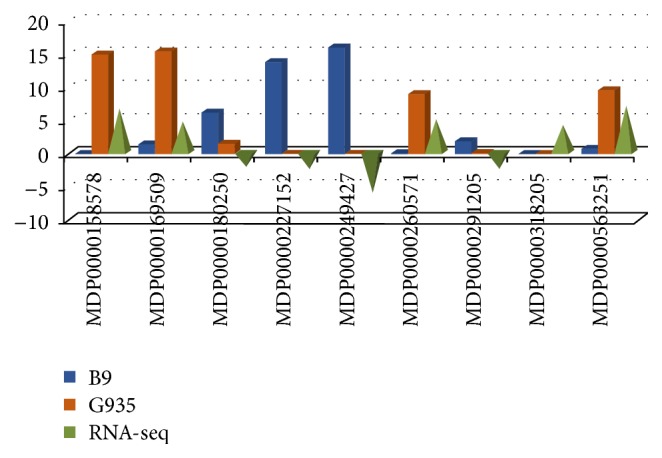
Validation of differential expression patterns for selected DEGs by qRT-PCR. Validation of the expression patterns for selected DEGs using qRT-PCR. Gene reference numbers were shown on the *x*-axis; blue and red bars show the relative expression level in the roots of B.9 and G.935 from qRT-PCR experiment; the green pyramid indicates the values of Log_2_FC from RNA-seq data; the upward direction represents the higher transcript level in the roots of G.935 and downward direction indicates lower transcript level in G.935. Values on the *y* axis denote the relative expression level for qRT-PCR and the Log_2_FC values from RNA-seq data set.

The expression patterns for nine DEGs identified by RNA-seq based comparative transcriptome analysis were validated by an independent approach of qRT-PCR (Figure 5). Gene specific primer sets, reference numbers in the apple genome database, and the RNA-seq data are shown in Supplementary File number 2. Although RNA-seq and qRT-PCR utilize different algorithms in quantifying their expression levels and huge variation of transcript abundance between two cultivars exist for some selected genes, most of the tested DEGs showed the consistency in dynamic gene expression patterns (Figure 4).

## 4. Discussion

In this study, the comparative analysis of the transcriptomes of two apple rootstock cultivars, a susceptible B.9 and a resistant G.935, under no pathogenic pressure, uncovered a wide spectrum of DEGs that were annotated as genes related to the plant host defense system. Previously, contrasting resistance phenotypes were demonstrated to exist between these two cultivars when they were challenged by the soilborne pathogen of* P. ultimum* [11]. Interestingly, the current data set indicated that a preformed defense system exists in the root tissues of resistant cultivar G.935, but not susceptible B.9. Notably, because a synchronized tissue culture procedure was performed in preparing the plants with equivalent developmental stage between these two cultivars and these plants were maintained under identical conditions (light cycle, watering schedule, and environmental temperature) with minimized stress, such profound differences at the transcriptome level seem unlikely to be caused by factors other than genetics.

Intraspecific gene expression variation, or expression level polymorphism (ELP), is known to be a common phenomenon [32–34]. Among rice germplasm accessions, a strong correlation was observed between phenotypes partially resistant to rice pathogen* Magnaporthe oryzae* and a preformed proactive defense system [12]. Higher gene expression levels and enzymatic activities related to defense responses were also observed in uninoculated kernels of disease resistant maize lines, interpreted as conferring a major readiness to the pathogen attack [13]. In another study, a large number of differentially expressed defense-related genes were identified between two near-isogenic lines of* Brassica rapa* at 0-hour postinoculation (hpi), that is, before encountering the pathogen* Plasmodiophora brassicae* [32]. Therefore, preformed defense due to constitutive expression of defense-related genes may commonly exist, although the underlying mechanism(s) causing such sustained and elevated expression of defense-related genes has not been well elucidated.

In an attempt to explain the mechanism potentially regulating preformed defense, Vergne et al. [12] proposed the “leakage” theory, in that an oversensitive regulator upstream of resistance pathways genes may be constantly agitated by some environmental factor(s) and that overactivated defense pathways would then lead to enhanced levels even in the absence of infection. Alternatively, a weak or “loss of function” allele for a “susceptibility” gene, or a negative regulator of resistance, upstream of a signal transduction pathway may result in wide-ranging transcriptome reprogramming. The broad-spectrum resistance from the recessive alleles of the barley MLO gene, a negative regulator of disease resistance, was believed to cause the constitutive expression of some disease resistance genes, even causing spontaneous HR-like cell-death [35, 36]. Specific to plant roots, the possibility exists, although perhaps less likely, that the unintentional introduction of an endophyte or even exterior biological or abiotic factor in the soil could trigger differential regulation of plant defense system genes in different genotypes. Recently it was reported that activation of pathways of secondary metabolism, which is involved in plant defense in the root of grapevine, was likely resulting from the colonization by endophytic bacteria [37].

Wide-spread and constitutive activation of multiple aspects of the plant defense system seems to be contradictory to the fitness cost for a plant species, as induced defense responses are generally believed to save energy under a pathogen-free environment. Nevertheless, it was also proposed by van Hulten et al. [38] that the benefits of priming-mediated resistance could outweigh its energy cost, assuming a high and constant pressure from the pathogen. This assumption could possibly apply to the root of a perennial plant, like apple, where the cumulative soilborne necrotrophic pathogens in the soil from long-life cycle fruit trees are a constant threat to the health of root system.

In the induced defense response, plants use discrete hormone balances and fine-tuned crosstalk to tailor specific reactions toward different types of attacking pathogens. It is well established that biosynthesis and signaling of ethylene (ET) and jasmonic acid (JA) are essential for plant defense responses toward necrotrophic pathogens [17, 18, 39–41]. Transcription factors (TFs) are the master-control proteins for swift transcriptional regulation during plant-pathogen interactions [26, 27]. For example, JA-inducible R2R3-MYB (myeloblastosis oncogene), ERF, and WRKY (containing signature WRKY amino acid residues) are known to be the key regulators in activating the phenylpropanoid biosynthetic pathway and expression of resistance genes [42, 43]. From the current data set, constitutive expressions for several defense hormone biosynthesis genes, and defense implicated TFs, were shown to have elevated transcript abundance in the root of the disease resistant cultivar G.935 as compared to that of the susceptible B.9 cultivar in the absence of pathogens (this paper). Also, it seems that different members from the same gene families, for example, the ACS gene family, are functioning in either preformed defense system or inducible defense activation [44, 45].

R protein encoding genes represent one of the groups with large number of DEGs identified from the present study. R proteins typically contain nucleotide-binding (NB) and leucine-rich repeat (LRR) domains and belong to a class of proteins known as NB-LRRs. These proteins can be further divided into two main groups based on their N-terminal domains [1, 46–48]. The first group possesses an N-terminal domain with sequence similarity to the intracellular signaling domains of the Drosophila Toll and mammalian interleukin- (IL-) 1 receptors and is referred to as TIR-NB-LRRs or TNLs. The second group is collectively known as CC-NB-LRRs or CNLs, based on the presence of a predicted N-terminal coiled-coil domain in some, but not all, members of this class. The tobacco mosaic virus resistance gene, N, encodes a TIR-NB-LRR class of resistance protein [49]. Although the repertoires of the large number of NB-LRR genes can be predicated based on increasing available genome sequences, the respective differential regulation patterns of these R-genes in different tissues and between specific genotypes within a species are very limited. Our data set indicated that a two to one ratio of R-genes exhibited higher levels of expression in G.935 than those with lower expression levels, as compared to the same genes of susceptible cultivar B.9. The large number of identified R-genes may be partly due to capability of extensive expression variations at the genomewide scale by RNA-seq technology. Nevertheless, all identified DEGs, using −2 < Log_2_FC > 2 as cutoff, represent only about 3% of the apple genomes based on the predicted number of gene models; and defense-related genes are a small portion of all DEGs.

Many questions remain to be answered: What is the “central switch” upstream of defense system that turns on the numerous defense-related genes? Is epigenetic alteration among genotypes within a species a possible mechanism impacting the transcriptome? [50–52] Admittedly, the observations from the current study are limited to the difference at the transcript level; therefore, we must ask whether the elevated transcript level corresponds to enrichment of, respectively, encoded proteins. Are there any detrimental effects with respect to other aspects of development of the resistant G.935, due to the observed constitutive expression of defense-related genes? Overall the results from the current study provide a comprehensive overview into the root of a resistant and susceptible apple plant and offer a novel perspective to evaluate the roles of preformed defense to soilborne pathogens. Some of the identified genes, particularly those functioning upstream of the defense activation process, will be tested in further work to determine their robustness across rootstock germplasms with defined resistance traits; the nucleotide sequences of tested genes should make possible the development of molecular tools useful for breeders, to efficiently and accurately incorporate the resistance traits into new generations of apple rootstocks.

## 5. Conclusion

Although intraspecific gene expression level polymorphism (ELP) is a common phenomenon, it was somewhat unexpected that large numbers of defense-related genes and/or disease resistance genes were differentially regulated in the absence of pathogen infection in the roots of two apple rootstock genotypes. The identified DEGs encode proteins that encompass multiple functional groups in defense response, such as PRR for pathogen detection, proteins for defense hormone biosynthesis and signaling, TFs with known roles in defense activation, enzymes in secondary metabolism, and various classes of R proteins. Although numerous different allelotypes may exist for most apple genes due to high level heterogeneity of the apple genome [53], the breadth and the intensity of the differentially expressed, defense-related genes in the root tissue of the resistant cultivar of G.935 deserve additional careful experimentation. Better understanding of preformed defense, and of its potential to contribute to an overall disease resistance phenotype in a perennial fruit tree, could present novel opportunities for future crop improvement.

## Supplementary Material

The detailed information of identified DEGs were categorized in different groups; numbers of mapped read, Log_2_FC and associated p values based on statistical analysis, and detailed information of annotated function are listed in Supplementary file number 1. Sequences information of gene specific primer sets which were used to validate the expression patterns of selected DEGs, and their reference numbers in the apple genome database are listed in Supplementary file number 2.

## Figures and Tables

**Table 1:** Illumina reads and alignment to apple gene models.

Biological replicates	Number of fastq sequences	Overall alignment rate (percentage)
B.9-1.fastq	28327388	44.61
B.9-2.fastq	33356252	49.23
B.9-3.fastq	25790809	52.76

G.935-1.fastq	26469741	47.64
G.935-2.fastq	18354709	51.71
G.935-3.fastq	23359343	46.89

**Table 2:** DEGs with annotated function in defense hormone biosynthesis and signaling.

Apple gene model^a^	Functional annotations^b^	Log_2_FC^c^
*Ethylene (ET) biosynthesis*		
MDP0000286210	ACS 7-like	+2.0
MDP0000413933	ACS 8-like	+2.1
MDP0000282901	ACO 4-like	+2.1
MDP0000593536	ACO homolog	+2.1
MDP0000473933	ACO 1-like	+2.9

*Jasmonic acid (JA) biosynthesis*		
MDP0000281525	Linoleate 13S-lipoxygenase 2-1	+3.0
MDP0000753547	Linoleate 13S-lipoxygenase 2-1	+2.4
MDP0000154928	Lipoxygenase	−2.5

*ET/JA signaling*		
MDP0000297771	ERF 4	+4.0
MDP0000262710	ERF 027-like	+2.8
MDP0000128326	ERF RAP2-11-like	+2.4
MDP0000246808	ERF 1B-like	−2.1
MDP0000447570	ERF 1B-like	−2.2
MDP0000825712	Topless-related protein 4-like	+2.6
MDP0000313085	Topless-related protein 1-like isoforms X3	−2.2

*Gibberellin (GA) biosynthesis and signaling*		
MDP0000769340	GA receptor GID1	+5.2
MDP0000682570	GA receptor GID1	+3.6
MDP0000161639	GA receptor GID 1C-like	+2.3
MDP0000431628	DELLA protein RGL1-like	+2.5
MDP0000126248	GA 2-beta-dioxygenase 7-like	+2.4
MDP0000226405	GA 2-beta-dioxygenase 2-like	−2.1

*Abscisic acid biosynthesis*		
MDP0000300259	ABA 8′-hydroxylase 3-like	+2.1
MDP0000326412	ABA 8′-hydroxylase 2	+3.7

*Cytokinin biosynthesis*		
MDP0000318353	Cytokinin dehydrogenase 1-like	+2.6

^a^Apple gene model is based on available Malus_x_domestica.v3.0.a1_gene_set at https://www.rosaceae.org. ^b^Function annotation is based on the blastX search against NR database in GenBank. ^c^Variations of transcript abundance are based on the comparison between B.9 and G.935 and expressed as Log_2_fold change; + indicated higher transcript level in the root of G.935 over that in B.9; **−** indicated the opposite.

**Table 3:** DEGs encoding proteins with annotated function of pattern recognize receptor (PRR).

Apple gene model^a^	Functional annotations^b^	Log_2_FC^c^
*Chitin elicitor receptor kinase 1- (CERK1-) like receptor*		
MDP0000182108	lysM domain receptor-like kinase 3	+3.13
MDP0000137744	lysM domain receptor-like kinase 3	+2.76
MDP0000225637	lysM domain receptor-like kinase 4	+3.10
MDP0000172006	lysM domain receptor-like kinase 4, isoform X1	+2.0

*BRASSINOSTEROID INSENSITIVE1-associated receptor kinase 1-like receptor*		
MDP0000651862	BAK1	+6.2
MDP0000423334	BAK1	+5.5

*Wall associated kinase- (WAK-) like receptor*		
MDP0000278145	WAK-like protein	−5.63
MDP0000311732	WAK-like protein	−2.45
MDP0000281090	Putative WAK-like 16	−2.60
MDP0000426154	Putative WAK-like 16	−2.67
MDP0000656197	Putative WAK-like 16	−3.02
MDP0000169245	Putative WAK-like 16	−2.40
MDP0000281090	Putative WAK-like 16	−3.0
MDP0000837936	Putative WAK-like 16	−2.05
MDP0000354964	Putative WAK-like 16	+4.12
MDP0000276990	Putative WAK-like 16	+2.31
MDP0000167101	WAK4-like	−2.14
MDP0000465085	WAK3-like	+2.04
MDP0000170906	WAK2-like	+2.10
MDP0000354964	WAK2-like	+4.12
MDP0000153539	WAK-like 8 isoform X2	+2.8

^a^Apple gene model is based on available Malus_x_domestica.v3.0.a1_gene_set at https://www.rosaceae.org. ^b^Function annotation is based on the blastX search against NR database in GenBank. ^c^Variations of transcript abundance are based on the comparison between B.9 and G.935 and expressed as Log_2_Fold Change; + indicated higher transcript level in the root of G.935 over that in B.9; **−** indicated the opposite.

**Table 4:** DEGs encoding lectin receptor kinases and glutamate receptors.

Apple gene model^a^	Functional annotations^b^	Log_2_FC^c^
*L-type lectin-domain containing receptor kinase*		
MDP0000142818	L-type lectin-RLK; IV.1-like	+5.5
MDP0000226962	L-type lectin-RLK; L-type lectin-RLK X.1	+2.5
MDP0000265486	L-type lectin-RLK; IX.1-like [*Malus domestica*]	+5.4
MDP0000305188	L-type lectin-RLK; IX.1-like	+4.1
MDP0000176219	L-type lectin-RLK; IX.1-like	+4.0
MDP0000199947	L-type lectin-RLK, IX.1-like	+2.1
MDP0000130183	L-type lectin-RLK, S.1-like	−3.3
MDP0000133353	L-type lectin-RLK, S.4-like	+2.6
MDP0000200920	L-type lectin-RLK, S.4-like	−4.0
MDP0000145928	L-type lectin-RLK, VIII.2-like	−3.2

*G-type lectin S-receptor-like serine/threonine-protein kinase*		
MDP0000297547	G-type lectin SRK, At1g11410	+3.9
MDP0000303074	G-type lectin SRK, At1g11410	+3.7
MDP0000930643	G-type lectin SRK, At1g11410 isoform X1	+6.0
MDP0000296847	G-type lectin SRK, At1g11410 isoform X2	+2.0
MDP0000263753	G-type lectin SRK, At1g61500	+2.2
MDP0000278380	G-type lectin SRK, At1g61500	+2.5
MDP0000212928	G-type lectin SRK, At1g61500	+2.4
MDP0000550024	G-type lectin SRK, At1g61550	+2.5
MDP0000172360	G-type lectin SRK, At1g61550	−2.3
MDP0000204472	G-type lectin SRK, At1g61550	−2.9
MDP0000936164	G-type lectin SRK, At1g67520	+6.0
MDP0000284325	G-type lectin SRK, At2g19130	+3.0
MDP0000654452	G-type lectin SRK, At2g19130	+2.4
MDP0000314519	G-type lectin SRK, At4g27290	+2.3
MDP0000189422	G-type lectin SRK, At4g27290	+2.1
MDP0000281186	G-type lectin SRK, At4g27290 isoform X1	−2.6
MDP0000213485	G-type lectin SRK, At4g27290 isoform X5	−2.3
MDP0000293905	G-type lectin SRK, RKS1	2.6
MDP0000314952	G-type lectin SRK, RKS1, isoform X4	−2.9
MDP0000288873	G-type lectin SRK, RLK1	+4.7
MDP0000189901	G-type lectin SRK, RLK1	+4.7
MDP0000217223	G-type lectin SRK, RLK1	+3.2
MDP0000627319	G-type lectin SRK, RLK1	+3.2
MDP0000322228	G-type SRK, RLK1	+3.2
MDP0000310978	G-type lectin SRK, SD1-1	−4.4

*Glutamate receptor*		
MDP0000176838	Glutamate receptor 1.3-like	+2.2
MDP0000314708	Glutamate receptor 2.2-like	+2.3
MDP0000450755	Glutamate receptor 2.2-like	−3.1
MDP0000771031	Glutamate receptor 2.7-like	+2.7
MDP0000318184	Glutamate receptor 2.7-like	+2.3
MDP0000220955	Glutamate receptor 2.8-like	+4.8
MDP0000291967	Glutamate receptor 2.8-like	−2.6
MDP0000228982	Glutamate receptor 2.8-like	−2.9
MDP0000255245	Glutamate receptor 2.8-like	−4.4
MDP0000154359	Glutamate receptor 2.8-like	−4.7
MDP0000307622	Glutamate receptor 3.4-like	+5.2
MDP0000199967	Glutamate receptor 3.4-like	+4.9
MDP0000307121	Glutamate receptor 3.4-like	+4.2
MDP0000487438	Glutamate receptor 3.6-like	+2.0

^a^Apple gene model is based on available Malus_x_domestica.v3.0.a1_gene_set at https://www.rosaceae.org. ^b^Function annotation is based on the blastX search against NR database in GenBank. ^c^Variations of transcript abundance are based on the comparison between B.9 and G.935 and expressed as Log_2_fold change; + indicated higher transcript level in the root of G.935 over that in B.9; **−** indicated the opposite.

**Table 5:** DEGs encoding other receptor kinases and receptor-like proteins.

Receptor groups	Number of DEGs with higher transcript abundance in G.935	Number of DEGs with lower transcript abundance in G.935
LRR receptor-like serine/threonine-protein kinase	68	37
Receptor-like protein kinase	42	14
Receptor-like protein	36	8
Cysteine-rich receptor-like protein kinase	12	5

**Table 6:** DEGs with the annotated function of oxidation and reduction processes.

Gene model^a^	Functional annotations^b^	Log_2_FC^c^
*Glutathione S-transferase*		
MDP0000183814	Glutathione S-transferase	+2.1
MDP0000138709	Glutathione S-transferase	−2.0
MDP0000161008	Glutathione S-transferase parA	−2.2
MDP0000175866	Glutathione S-transferase parA	−2.4
MDP0000276730	Glutathione S-transferase U8-like	−2.8
MDP0000252292	Glutathione S-transferase F12-like	−4.4

*Peroxidase*		
MDP0000203927	Glutathione peroxidase 8 isoform X1	+4.8
MDP0000455043	Peroxidase A2-like, partial	+2.4
MDP0000269302	Peroxidase 60-like	+2.2
MDP0000451182	Peroxidase 66-like	+2.0
MDP0000493703	Peroxidase N1-like	−2.2
MDP0000283650	Peroxidase	−2.3
MDP0000684133	Peroxidase N1-like	−2.4
MDP0000233961	Peroxidase A2-like	−2.5
MDP0000668551	Peroxidase N1-like	−2.5
MDP0000166657	Peroxidase 51-like	−2.6
MDP0000338065	Glutathione peroxidase isoform X1	−2.9
MDP0000173751	Peroxidase 59-like	−2.9
MDP0000206714	Peroxidase N1-like	−3.1
MDP0000298916	Peroxidase 4-like isoform X1	−3.1
MDP0000494230	Peroxidase N-like	−3.9
MDP0000172233	Peroxidase 50-like	−4.2
MDP0000212661	Glutathione peroxidase isoform X1	−5.4
MDP0000580571	Peroxidase N-like	−5.4

^a^Apple gene model is based on available Malus_x_domestica.v3.0.a1_gene_set at https://www.rosaceae.org. ^b^Function annotation is based on the blastX search against NR database in GenBank. ^c^Variations of transcript abundance are based on the comparison between B.9 and G.935 and expressed as Log_2_fold change. + indicated higher transcript level in the root of G.935 over that in B.9; **−** indicated the opposite.

**Table 7:** DEGs encoding pathogenesis-related proteins and cell wall metabolism enzymes.

Apple gene model^a^	Functional annotations^b^	Log_2_FC^c^
*Pathogenesis-related protein 1-like*		
MDP0000202342	Pathogenesis-related protein 1-like	+5.5
MDP0000144351	Pathogenesis-related protein 1-like	+3.8

*Chitinase family protein*		
MDP0000202342	Chitinase family protein	−3.2
MDP0000144351	Chitinase family protein	−2.9
MDP0000171646	Acidic endochitinase SE2-like	−2.6
MDP0000282918	Acidic mammalian chitinase-like	−2.1

*Thaumatin-like protein*		
MDP0000916930	Thaumatin-like protein 1a	+4.9
MDP0000205389	Thaumatin-like protein 1a	+2.9
MDP0000552328	Thaumatin-like protein 1a	+2.3
MDP0000218699	Thaumatin-like protein 1a	+2.1
MDP0000293451	Thaumatin-like protein 1-like	+2.1
MDP0000124752	Thaumatin-like protein 1a	−3.3

*Glucan endo-1,3-beta-glucosidase*		
MDP0000908326	Glucan endo-1,3-beta-glucosidase 9-like	−2.2
MDP0000231628	Glucan endo-1,3-beta-glucosidase 4-like	−2.0
MDP0000237685	Glucan endo-1,3-beta-glucosidase 12-like	−5.8

*Pectin esterase inhibitor*		
MDP0000188907	Pectin esterase inhibitor 16	+3.1
MDP0000161463	Pectin esterase inhibitor 35	+3.0
MDP0000299506	Pectin esterase inhibitor 24	+2.5
MDP0000611653	Pectin esterase inhibitor 24	+2.4
MDP0000299884	Pectin esterase inhibitor 24	+2.2
MDP0000503248	Pectin esterase inhibitor family protein	+3.0

*Polygalacturonase*		
MDP0000845683	Polygalacturonase	+2.3
MDP0000845685	Polygalacturonase	+2.6

*Cellulose synthase-like protein *		
MDP0000208533	Cellulose synthase-like protein E2	+4.1
MDP0000172542	Cellulose synthase-like protein G3	+2.4
MDP0000223480	Cellulose synthase-like protein E1	−2.1

^a^Apple gene model is based on available Malus_x_domestica.v3.0.a1_gene_set at https://www.rosaceae.org. ^b^Function annotation is based on the blastX search against NR database in GenBank. ^c^Variations of transcript abundance are based on the comparison between B.9 and G.935 and expressed as Log_2_fold change. + indicated higher transcript level in the root of G.935 over that in B.9; **−** indicated the opposite.

**Table 8:** Genes encoding proteins functioning in secondary metabolism.

Gene families^a^	Number of DEGs with higher transcript abundance in G.935	Number of DEGs with lower transcript abundance in G.935
UDP-glucose flavonoid 3-O-glucosyltransferase 7-like	1	1
4-Hydroxybenzoategeranyl transferase 2-like	1	0
Phenylalanine ammonia-lyase class 1-like	1	0
Phytoene synthase 2, chloroplastic-like	2	0
Squalene monooxygenase-like	2	0
Isoflavone reductase homolog	1	4
3-Ketoacyl-CoA synthase-like	1	5
Cinnamoyl-CoA reductase 1-like	3	3
Geraniol 8-hydroxylase-like	0	4
Caffeic acid 3-O-methyl transferase-like	3	2
Flavonoid 3′-monooxygenase-like	4	0
UDP-glycosyl transferase-like	14	9
Beta-glucosidase	14	9

^a^Function annotation is based on the blastX search against NR database in GenBank.
