# Supplementary material for: Comparative Transcriptome Analysis Reveals a Preformed Defense System in Apple Root of a Resistant Genotype of G.935 in the Absence of Pathogen
Source: Int J Plant Genomics. 2017 Mar 30;2017:8950746. doi: 10.1155/2017/8950746 (PMC5390597; doi:10.1155/2017/8950746)
Supplement: Supplementary file 1 — The detailed information of identified DEGs were categorized in different groups; numbers of mapped read, Log2FC and associated p values based on statistical analysis, and detailed information of annotated function are listed in Supplementary file number 1. Sequences information of gene specific primer sets which were used to validate the expression patterns of selected DEGs, and their reference numbers in the apple genome database are listed in Supplementary file number 2. [file 8950746.f1.zip › Supplementary file No.2.docx]

| Gene Number | Forward primer (5’-3’) | Reverse primer (5’-3’) |
| --- | --- | --- |
| MDP0000158578 | GTGGTAGTGAGGGAAGTTGATT | AGCTAGGCCCTTTGTTATTCTC |
| MDP0000169509 | CAACACGTGCCTTCTGTTAT | TTTGCGAGATTGGCCTT |
| MDP0000180250 | TGATACAAAGCCTGAGCTTGA | AGCAGAAGAGCATGCAGTAG |
| MDP0000227152 | CCTCAACTCATTGACGCTTTAC | GGGAATAGCTGATGACCCTATC |
| MDP0000249427 | TCCTCCTCCTCCTTCTTCTTC | CCGGATCATCGCCATCAAT |
| MDP0000260571 | GCAGTCGTTTAGAGGTGGTT | GGTCTCGTAGTTGCTGAATACC |
| MDP0000291205 | GATTATGAGATGGAGTTTCCC | GAA AAG CCA TTT AGG ATG GC |
| MDP0000318205 | CTTAAAGAGAGGCTTAGTCG | ATT GAG TAC GCT TCT ATC TC |
| MDP0000563251 | AACCACCATCACCATCATCTC | GCGTGTCTACACCGCTAAA |
| MDP0000752428  (*MdActin*) | GTC GTA CTA CTG GTA TCG TT | TCA TAG TCA AGA GCA ATG TA |

Supplementary file No. 2. Primer sequences used for qRT PCR validation
